# Primary pilocytic astrocytoma of the cerebellopontine angle in pediatric patient with neurofibromatosis type 1: a case report

**DOI:** 10.1186/s41984-022-00168-8

**Published:** 2022-08-31

**Authors:** Zaitun Zakaria, Raja Zubaidah Raja Mohd Rasi, Noor Azman A. Rahman

**Affiliations:** 1 Department of Neurosurgery, Hospital Sultanah Aminah Johor Bahru, Jalan Persiaran Abu Bakar Sultan, 80100 Johor Bahru, Johor Malaysia; 2 Department of Neurosciences, School of Medical Sciences, Universiti Sains Malaysia, 16150 Kubang Kerian, Kota Bharu, Kelantan Malaysia; 3 Hospital Universiti Sains Malaysia, Universiti Sains Malaysia, Health Campus, Jalan Raja Perempuan Zainab 2, 16150 Kota Bharu, Kelantan Malaysia; 4 Department of Pathology, Hospital Sultanah Aminah Johor Bahru, Jalan Persiaran Abu Bakar Sultan, 80100, Johor Bahru, Johor Malaysia

**Keywords:** Cerebellopontine angle, Neurofibromatosis, Neurofibromas, Pilocytic astrocytoma, Schwannoma

## Abstract

**Background:**

Cerebellopontine angle tumor (CPA) in pediatrics is rare as compared to adults. We describe a case of pediatric pilocytic astrocytoma presented as a right CPA mass with a concurrent clinical diagnosis of neurofibromatosis type 1 (NF1).

**Case presentation:**

A 14-year-old boy with a newly diagnosed hypertension presented with a short history of headache and blurring vision. Neurological examination revealed bilateral papilloedema, partial right third nerve palsy and mild sensorineuronal hearing deficits. Skin examination identified multiple café au lait spots with cutaneous neurofibromas. Preoperative neuroimaging suggested the diagnosis of an extraaxial CPA mass consistent with meningioma, with obstructive hydrocephalus. A left ventriculoperitoneal shunt was inserted and the child was subjected for a suboccipital retrosigmoid approach for tumor resection. The histopathological features, however, were typical for pilocytic astrocytoma.

**Conclusions:**

A careful evaluation of the clinical presentation and radiological images of CPA lesions is necessary prior to surgical embarkment. To the best of our knowledge, this case is the first report of pilocytic astrocytoma in the CPA in pediatric with NF1.

## Introduction

Cerebellopontine angle (CPA) tumors in pediatrics are rare as compared to adults. The incidence was previously reported at 1%, more frequently benign (> 70%) than malignant and likely to occur on the left side [1]. Vestibular schwannoma (VS) and CPA meningioma are commonly found in pediatrics with neurocutaneous disorder such as neurofibromatosis type 2 (NF2) [2], rather than in NF1. Isolated VS may present earlier than the diagnosis of NF2 and tends to be larger (average size of 3.4 cm), more aggressive and hypervascular [3, 4].

CPA glioma is discussed in the literature as case reports [5]. Pilocytic astrocytomas were both reported in adult (1 case) [6] and pediatric (3 cases) [5, 7, 8]. One case of pilocytic astrocytomas arises from trigeminal [5], while the rest is from vestibular. Herein we present a primary pediatric CPA pilocytic astrocytoma with concurrent clinical diagnosis of NF1. To the best of our knowledge, there were no previous reports of similar cases.

## Case description

**Fig. 1:**
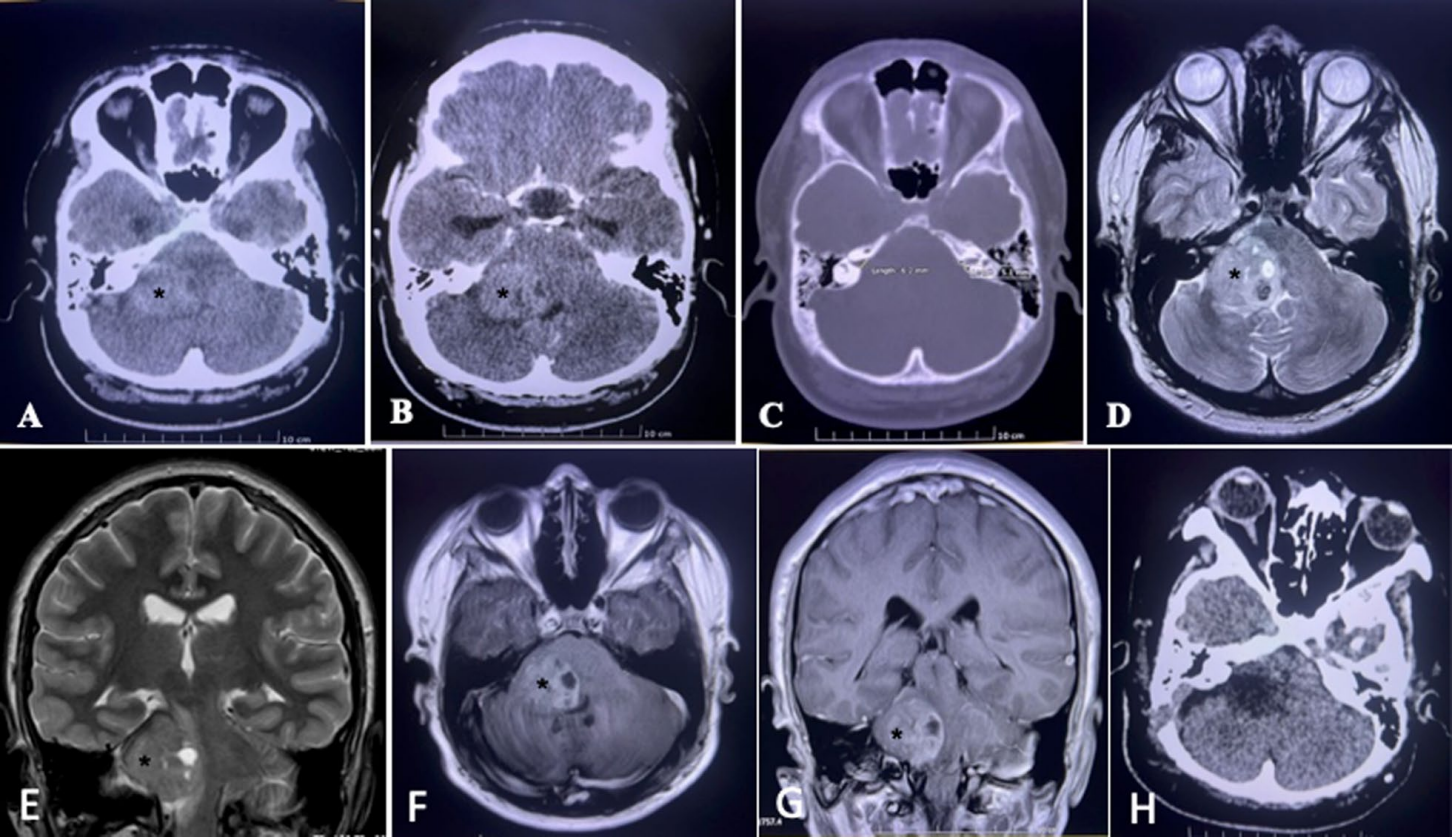
Radiological images. **A** An axial CT brain revealing a right hypodense CPA tumor (asterisk) that enhances following **B** contrast sequences, with **C** IAC enlargement. T2-weighted MR image demonstrating heterogeneous intensity lesions on **D** axial and **E** coronal sequence. **F**,** G** On the T1-weighted post gadolinium, a heterogenous enhancement is seen following gadolinium. **H** Day 1 postoperative axial CT brain showing resection cavity with partial relief of brain stem compression

A 14-year old boy with a newly diagnosed hypertension presented with a two-week history of right occipital headache that is radiating to the neck associated with blurring of vision. Neurological exams revealed bilateral papilloedema grade II, partial right third nerve palsy and sensorineuronal hearing deficits. The motor, sensory and cerebellar exams were unremarkable. Skin examination identified multiple café au lait spots over the thorax and abdomen with cutaneous neurofibromas. His father was diagnosed with NF1 in childhood. A noncontrast computed tomography (CT) showed a right extraaxial CPA lesion measuring 3.5 cm × 3.5 cm × 2.5 cm with obstructive hydrocephalus. There was a widening of the internal acoustic canal (IAC) (Fig. 1A–C). The patient was subjected to a left parietooccipital ventriculoperitoneal (VP) shunt and was discharged home a few days later. During a clinic visit, the brain magnetic resonance imaging (MRI) was reviewed, confirming a right IAC/CPA lesion, favoring benign VS and consistent with a new diagnosis of NF1 (Fig. 1D–G). Over the subsequent 4 months, he developed right facial palsy (House-Brackmann grade 3) and cerebellar ataxia. The family soon agreed to surgical intervention, however, the treatment was delayed due to a recent diagnosis of Coronavirus disease (COVID-19) stage 2 and poor control hypertension. He underwent a right retrosigmoid craniotomy approach three months after recovering from a COVID-19 infection.

Intraoperatively, after gentle medial retraction of the cerebellum, we encountered a solid with multiple cystic tumors over the cerebellopontine cistern. Intraoperative monitoring was utilized for trigeminal, facial and lower cranial nerve electromyography, motor and somatosensory evoked potential. The seventh nerve was pushed anterior and superiorly while the eighth cranial nerve was encased by the tumor. The trigeminal and lower cranial nerve was identified and preserved. The tumor was pale to yellowish in color, soft in consistency with moderate vascularity. The tumor center was debulked using a cavitron ultrasonic aspirator until the tumor capsule became mobile. A demarcated arachnoid cleavage was identified with interface between the tumor and cerebellum, middle cerebellar peduncle and part of the pontine region. The tumor abutting the pontomedullary junction was not removed to prevent neurovascular injury.

**Fig. 2:**
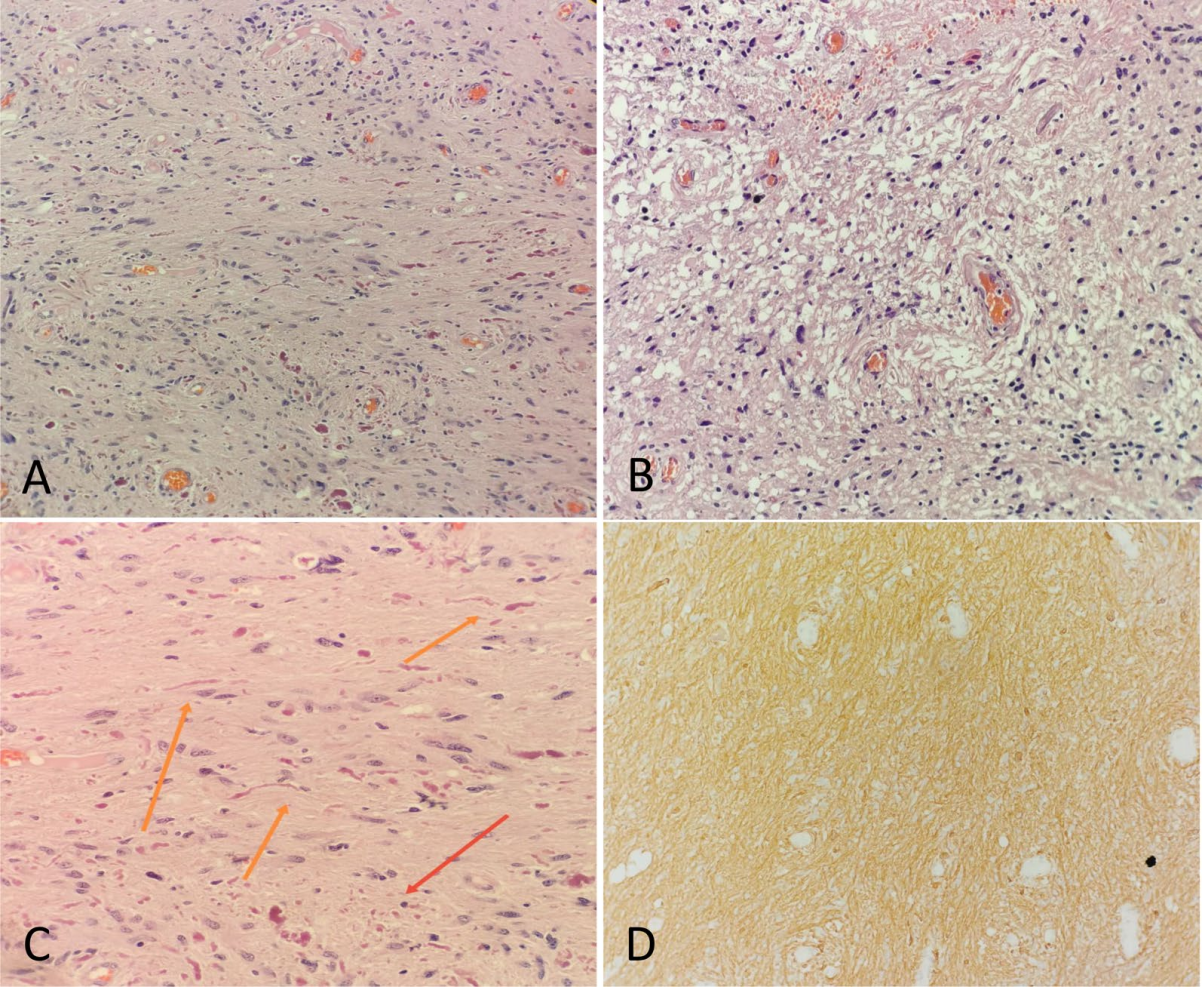
Hematoxylin–eosin stain. The tumor shows a biphasic appearance with **A** compact fibrillar area, and **B** loose microcystic area (× 10 magnification). **C** Rosenthal fibers (orange arrow) and eosinophilic granular bodies (red arrow) are present (× 20 magnification). **D** Immunohistochemical expression of positive glial fibrillary acidic protein (× 10 magnification)

Gross pathologic examination revealed fragments of pale to greyish tumor tissue. Microscopic examination revealed a biphasic appearance: the compact fibrillar area composed of elongated nuclei, bipolar piloid processes and Rosenthal fibers, and the loose microcystic cavity with oval-shaped nuclei. There were scattered eosinophilic granular bodies with observed hyalinised blood vessels. There was no mitosis or endothelial proliferation seen. Immunohistochemical stains were strongly positive for glial fibrillary acidic protein and negative for p53. The Ki67 index is less than 1%. These histopathological features were typical for pilocytic astrocytoma (Fig. 2). His postoperative course was complicated with worsening right facial palsy (house-brackmann grade 4) and reduced corneal reflex. He developed hospital-acquired pneumonia (HAP) requiring a course of antibiotic treatment and end up with tracheostomy.

## Discussion

The differentiation between extraaxial and exophytic extension of primary brain tumor into the CPA can be appreciated intraoperatively. The tumor located extraaxially has an arachnoid cleavage plane between the tumor and cerebellum, middle cerebellar peduncle, and the pons [5]. Other tumors that are not restricted to bona fide brain tumors include kaposiform hemangioendothelioma [9, 10], sarcomatous lesions such as angiosarcoma [11], alveolar soft part sarcoma and Ewing’s sarcoma/ primitive neuroectodermal tumor (PNET) family [10]. Meanwhile, the exophytic brain tumor arises from the brain parenchyma and protrude laterally. However, certain tumors such as medulloblastoma, ependymoma and atypical teratoid/rhabdoid tumors [10] may penetrate IAC causing enlargement of the internal auditory canal, hence mimicking an extraaxial tumor [7, 8].

Children with NF1 may develop a benign or malignant intracranial tumors. Tumors related to posterior fossa are cerebellar astrocytoma, medulloblastoma and brainstem gliomas [12–14]. The brainstem glioma is predominantly in the pons and 28% of children who were diagnosed with NF1 had a concurrent brainstem glioma [15]. To the best of our knowledge, this is the first report of pilocytic astrocytoma in the CPA in pediatric with NF1. The postoperative complication of HAP may have an association with his impaired lung function after COVID-19 infection.

## Conclusions

We presented the first pediatric case of right CPA pilocytic astrocytoma with a concurrent clinical diagnosis of NF1. A careful evaluation of preoperative MRI images of CPA lesions in such patient is necessary and to bear in mind other presumptive pathology prior to surgical embarkment.

## Data Availability

Not applicable.

## Abbreviations

CPA: Cerebellopontine angle
CT: Computed tomography
HAP: Hospital-acquired pneumonia
IAC: Internal acoustic canal
MRI: Magnetic resonance imaging
NF1: Neurofibromatosis type 1
NF2: Neurofibromatosis type 2
PNET: Primitive neuroectodermal tumor
VP: Ventriculoperitoneal
VS: Vestibular schwannoma
